# Supplementary material for: Mixed topological semimetals driven by orbital complexity in two-dimensional ferromagnets
Source: Nat Commun. 2019 Jul 18;10:3179. doi: 10.1038/s41467-019-10930-6 (PMC6639329; doi:10.1038/s41467-019-10930-6)
Supplement: Supplementary file 1 — Supplementary Information [file 41467_2019_10930_MOESM1_ESM.pdf]

Mixed topological semimetals driven by orbital complexity in two-dimensional ferromagnets

Niu et al.

SUPPLEMENTARY FIGURES

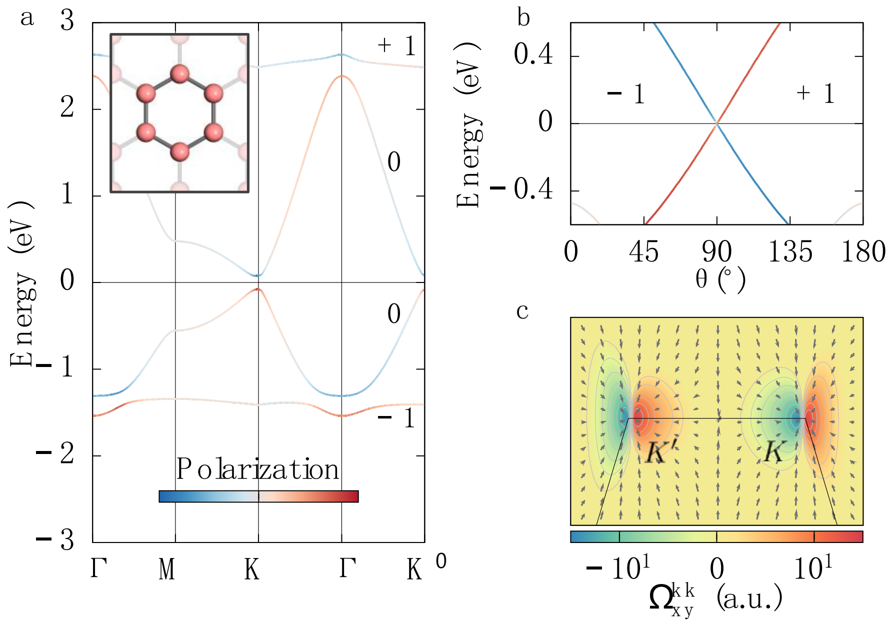

Supplementary Figure 1. **Mixed Weyl points in the planar model.** (a) Electronic band structure of the p-model without buckling for θ=85°, where bold integers indicate the individual Chern numbers of the bands. Colors indicate the polarization in terms of p_x-ip_y (blue) and p_x+ip_y (red) orbital character, and the planar honeycomb lattice is shown as inset. (b) Evolution of valence band top and conduction band bottom as function of the magnetization direction θ. Colors denote the orbital polarization as in (a), and the net Chern number of the occupied states is given in bold. (c) Distribution of the Berry curvature field for θ=90°, which uncovers two emergent mixed Weyl points of negative unit topological charge at K and K'.

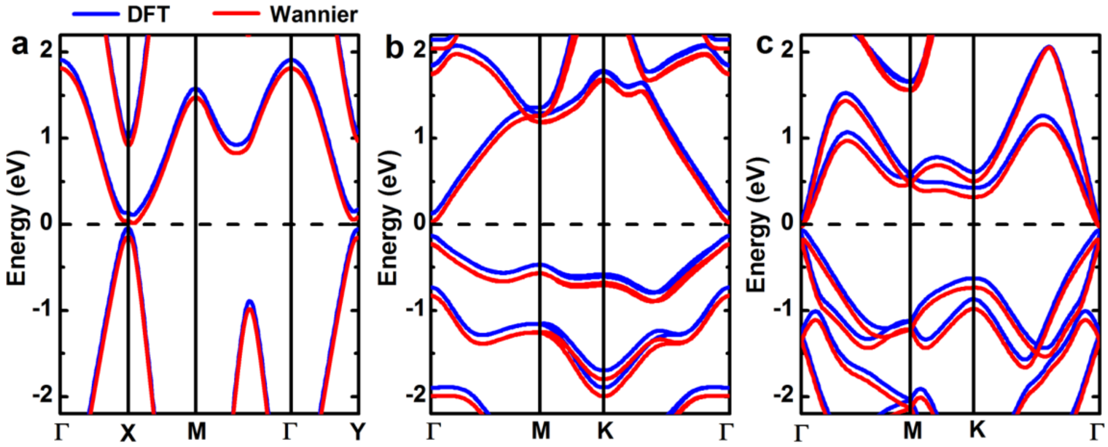

Supplementary Figure 2. **Band structures from first principles and from Wannier interpolation.** Comparison between interpolated and first-principles (DFT) band structures including spin-orbit coupling for (a) TlSe, (b) Na_3Bi, and (c) GaBi. The interpolated energy bands, which have been shifted down by 30 meV for visibility, are in excellent agreement with the first-principles bands in all cases.

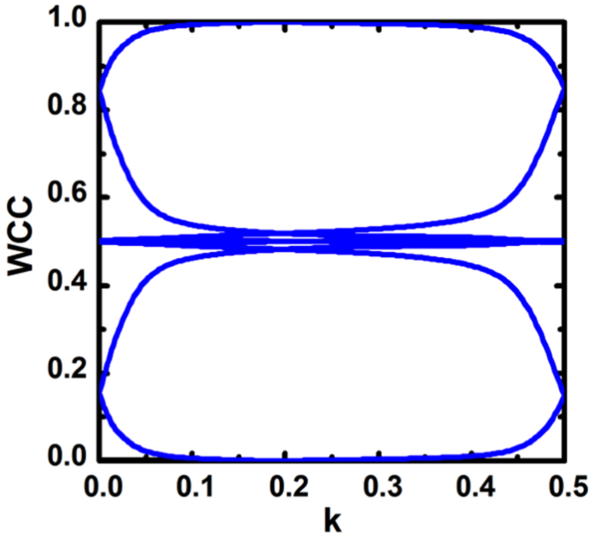

Supplementary Figure 3. **Wannier charge centers in magnetized TlSe.** For an applied exchange field of magnitude B = 0.1 eV and direction θ = 30º, the momentum evolution of the Wannier charge centers of TlSe reveals the topologically non-trivial nature of the system. Wannier charge centers are connected and there is no gap.

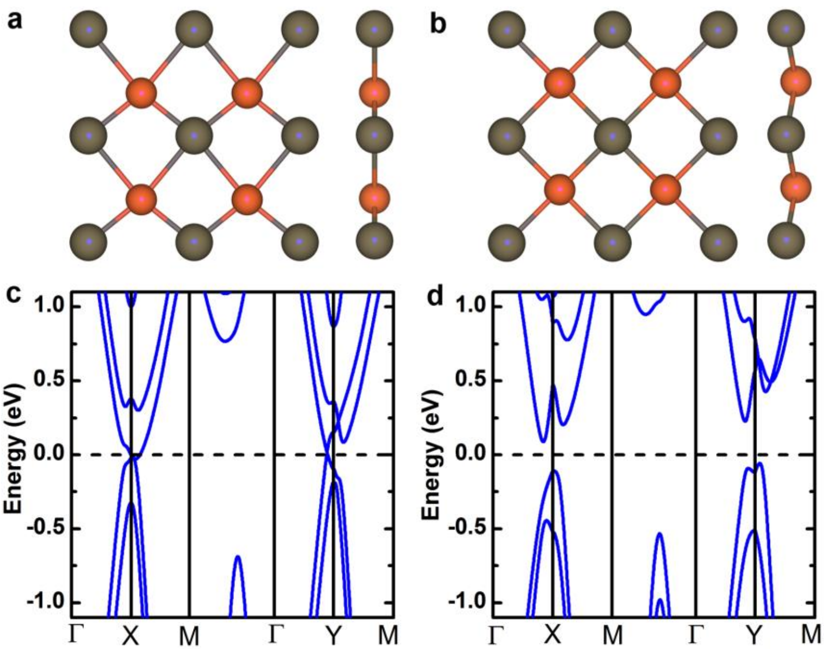

Supplementary Figure 4. **Role of mirror symmetry in TlSe.** (a,b) Top and side views of TlSe under distortions of the crystal lattice. While displacing the Se atom along an in-plane direction preserves the mirror symmetry with respect to the film plane, this symmetry is broken if Se is shifted out of the plane. (c,d) Electronic band structures of magnetized TlSe with an in-plane exchange field of magnitude B = 0.5 eV for the two corresponding structures shown in (a,b). The mixed Weyl points remain intact in the presence of the mirror symmetry but gap out as soon as this symmetry is broken.

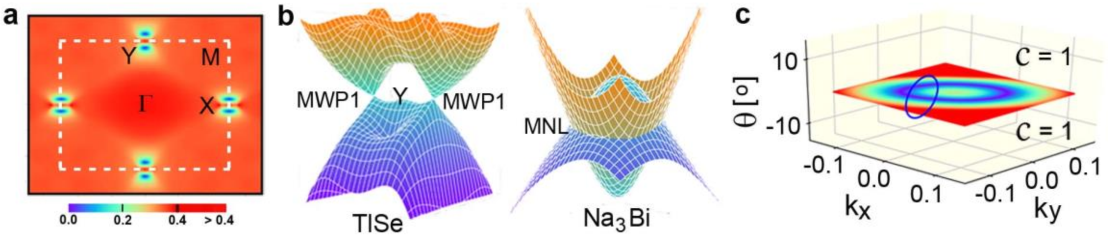

Supplementary Figure 5. **Mixed Weyl points and mixed nodal lines.** (a) variation of the direct band gap (in eV) throughout the Brillouin zone of the TlSe monolayer with an in-plane exchange field of magnitude B=0.5 eV. In the vicinity of X and Y, valence and conduction bands cross forming mixed Weyl points. (b) Three-dimensional illustration of the band dispersion in in-plane magnetized TlSe and perpendicularly magnetized Na_3Bi, revealing the existence of band crossings and nodal lines. (c) Microscopic distribution of the direct band gap in momentum space for Na_3Bi with B = 0.5 eV and $\theta = 0^o$. Carrying no topological charge as indicated by the constant Chern number, the emergent mixed nodal line is characterized by a non-trivial Berry phase in the complex phase space of k_x, k_y, and θ.

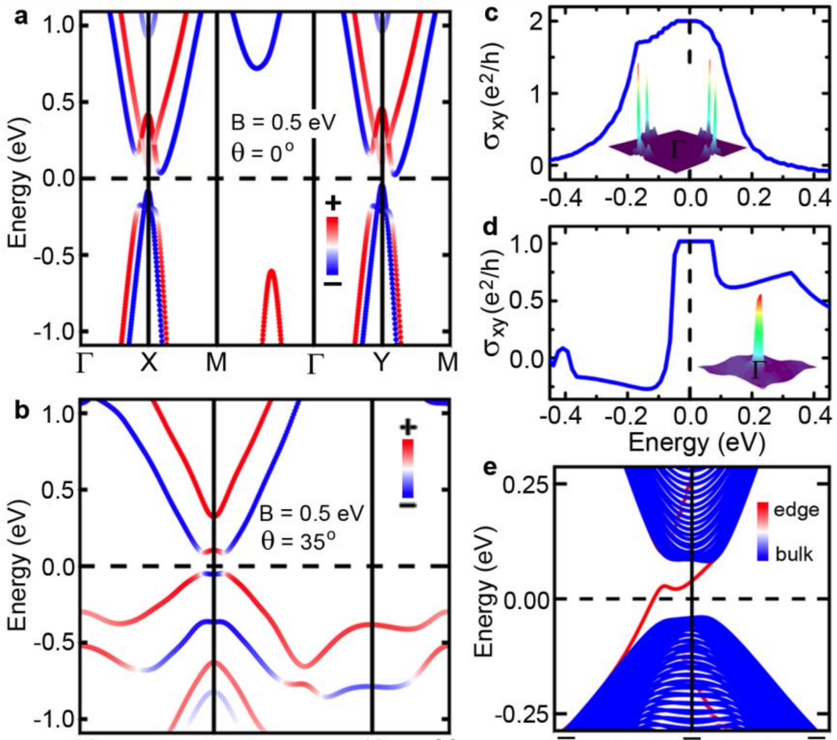

Supplementary Figure 6. **Quantum anomalous Hall phase in TlSe and Na_3Bi.** Spin-resolved band structures of (a) TlSe and (b) Na_3Bi monolayers with an applied exchange field of strength B = 0.5 eV and different magnetization directions θ as indicated. Colors represent the spin polarization perpendicular to the film plane. (c,d) Anomalous Hall conductivity σ_{xy} in TlSe and Na_3Bi as a function of the position of

the Fermi level E_F. Insets display the distribution of the momentum Berry curvature at the actual Fermi energy. (e) The quantum anomalous Hall phase in the Na_3Bi monolayer is accompanied by characteristic edge states as evident from the band structure of a one-dimensional ribbon of Na_3Bi with Bi-Na termination.

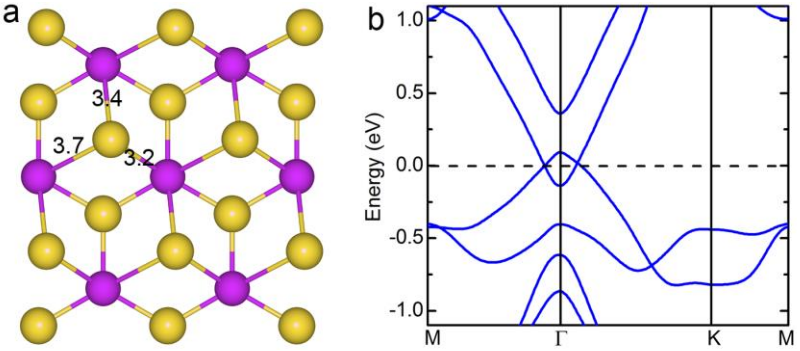

Supplementary Figure 7. **Mixed nodal line in Na_3Bi with rotation symmetry breaking.** (a) Top view of Na_3Bi under distortions of the crystal lattice. The numbers indicate the Bi-Na bond length in Å. The three-fold rotation symmetry is broken while shifting the Bi atom along an in-plane direction. However, the mirror symmetry survives. (b) Electronic band structures of magnetized Na_3Bi with an out-of-plane exchange field of magnitude B = 0.5 eV. The mixed nodal line remains intact.

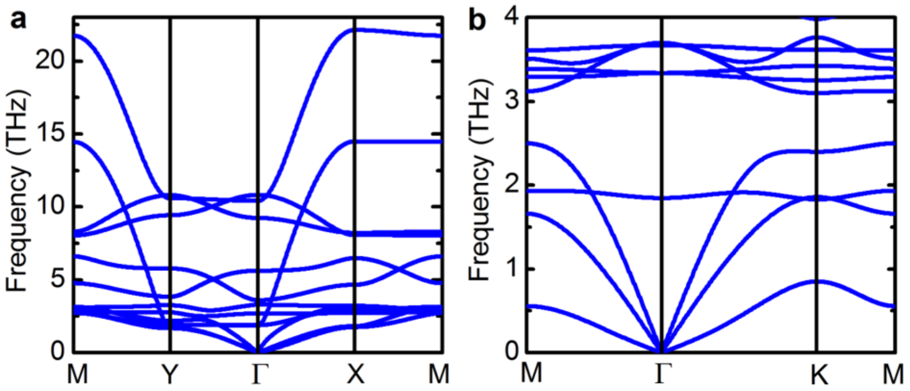

Supplementary Figure 8. **Stability of the ferromagnetic candidate materials.** The absence of modes with imaginary frequency in the phonon dispersion of (a) VOI_2 and (b) Na_2CrBi monolayers indicates that the structures are dynamically stable.

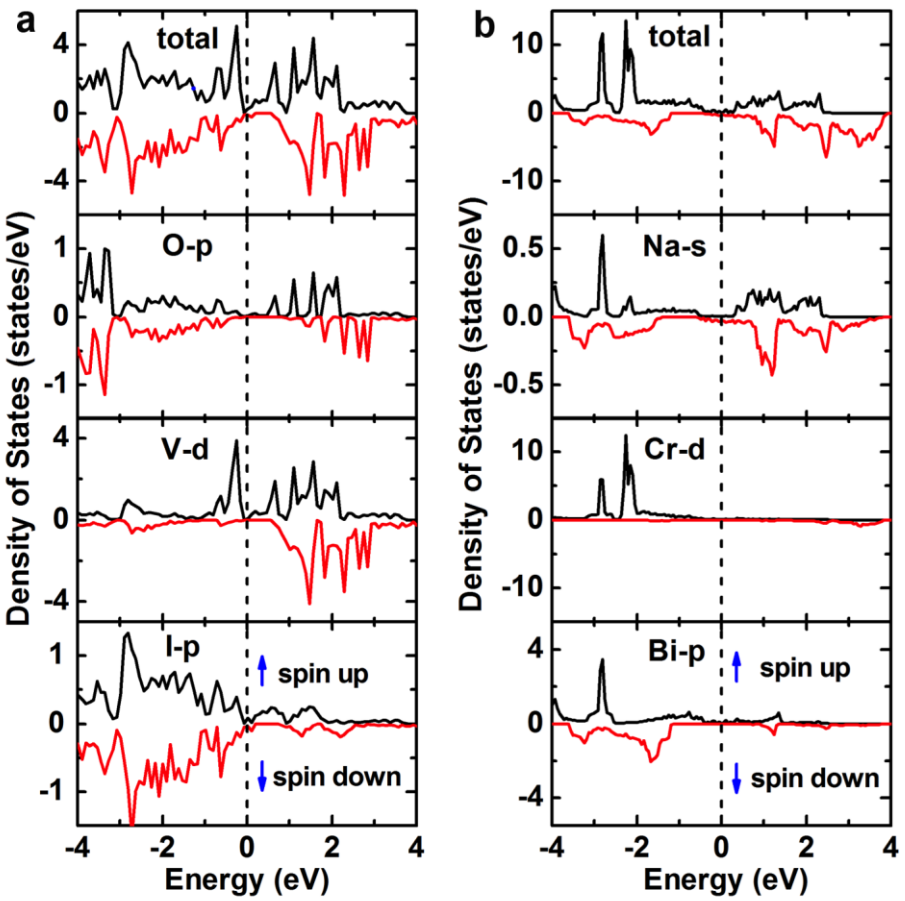

Supplementary Figure 9. **Magnetic properties of the ferromagnetic candidate materials.** Spin-polarized total and partial density of states for (a) VOI_2 and (b) Na_2CrBi monolayers, where positive and negative values represent spin-up and spin-down states, respectively. While the density of states in VOI_2 is mainly due to the *d*-states of Vanadium, the Cr-*d* states dominate the density for Na_3CrBi. Vertical dashed lines represent the Fermi level.

SUPPLEMENTARY NOTES

Supplementary Note 1 – Computational details for TlSe, Na_3Bi, and GaBi films

As reported recently, non-magnetic monolayers of TlSe[1], Na_3Bi[2], and GaBi[3] are topological crystalline insulators and/or topological insulators with large band gaps. In order to uncover the emergence of metallic points as the direction of an applied magnetic field is varied, we study the electronic structure of these systems based on density functional theory. Using the full-potential linearized augmented-plane-wave code FLEUR[4], we sample the Brillouin zone in the self-consistent calculations including spin-orbit coupling by a grid of 25×25×1 **k**-points and use a plane-wave cut-off of 4.0 $bohr^{-1}$. Subsequently, we constructed maximally-localized Wannier functions using the WANNIER90 code[5], where the frozen window extends up to 2 eV

above the Fermi energy. Based on the obtain tight-binding representation, we employ an efficient interpolation scheme that reproduces the first-principles electronic structure accurately as illustrated in Supplementary Figure 2.

Supplementary Note 2 – Role of broken mirror symmetry in TlSe

The monolayer of TlSe is known to host the two-dimensional topological crystalline insulator phase owing to the mirror symmetry of the underlying lattice. By introducing an additional exchange field with direction θ, which is not perpendicular to the film plane, we can break this symmetry operation. As shown in Supplementary Figure 3, the non-trivial topological crystalline insulator is realized even in the latter case if the exchange field is small enough. In analogy to the time-reversal-broken quantum spin Hall insulator[6], we refer to this phase as mirror-broken topological crystalline insulator in the main text. When increasing the exchange field, we can obtain the quantum anomalous Hall effect or the mixed Weyl semimetallic state, depending on the magnetization direction as discussed in the main text. Remarkably, the combination of time reversal and crystalline mirror symmetry is preserved for an in-plane magnetization, which guarantees the emergence of the mixed Weyl semimetal. To confirm this, we further consider perturbations of the crystal lattice by moving Se with respect to the Tl atoms either in the film plane or perpendicular to it, realizing two distinct cases of different symmetry. Supplementary Figure 4 shows that the mixed Weyl semimetal is not obtained if Se is shifted out of plane since this perturbation breaks the crystalline mirror symmetry and accordingly preserves not the combined symmetry of time reversal and mirror operation.

Supplementary Note 3 – Quantum anomalous Hall effect

In the considered materials, the band gaps are sensitive to both the magnitude B and the direction θ of the applied exchange field. As apparent from the phase diagrams discussed in the main text, topological phase transitions that are accompanied by a gap closing manifest in all systems. Depending on the exchange field, the quantum anomalous Hall effect with a quantized value of the transverse Hall conductivity σ_{xy} can be realized. Supplementary Figure 6 shows as an example the case of TlSe with B = 0.5 eV and θ = 0°, as well as Na_3Bi with B = 0.5 eV and θ = 35°, where the respective spin-resolved band structures clearly reveal the insulating character of these systems. In order to verify the topologically non-trivial nature of this gap, we calculate the anomalous Hall conductivity σ_{xy}, which amounts to a quantized value throughout the band gap as evident from Supplementary Figure 6 (c,d). The resulting quantum anomalous Hall phase is characterized by the finite Chern numbers C = 2 and C = 1 for TlSe and Na_3Bi, respectively, which is directly correlated with the appearance of chiral edge states as plotted in Supplementary Figure 6 (e).

Supplementary Note 4 – Two-dimensional ferromagnetic candidate materials

Demonstrating the dynamical stability of the considered candidate ferromagnets,

Supplementary Figure 8 shows the calculated phonon spectrum of VOI_2 and Na_2CrBi monolayers, where all phonon branches are positive over the Brillouin zone. The in-plane lattice constants of the rectangular VOI_2 film are a = 3.64 Å and b = 4.00 Å. The ground state is found to be ferromagnetic (favorable by 63 meV over the antiferromagnetic state), and the total magnetization of about 1 μ_B per unit cell is provided predominantly by the V-*d* states as illustrated in Supplementary Figure 9(a). Owing to the spin-orbit interaction, our evaluation of the magnetic anisotropy reveals that the magnetic moment aligns preferentially along an in-plane direction, which is 0.32 meV lower in energy than the out-of-plane orientation. The further electronic and topological investigations as discussed in the main text establish the existence of the mixed Weyl semimetal phase in VOI_2. In the case of Na_2CrBi, which we demonstrate in the main text to host a mixed nodal-line, the in-plane lattice constant is a = 4.92 Å. Based on calculations of the total energy, the magnetic ground state of this system is verified to be ferromagnetic (favored by 245 meV over the antiferromagnetic order). Primarily *d*-states of Cr contribute to the total magnetization of about 5 μ_B per unit cell as indicated in Supplementary Figure 9(b). Finally, the ground state is perpendicularly magnetized due to a colossal magnetic anisotropy energy of 7.8 meV.

References

1. Niu, C. *et al.* Two-dimensional topological crystalline insulator and topological phase transition in TlSe and TlS monolayers. *Nano Lett.* **15**, 6071–6075 (2015).
2. Niu, C. *et al.* Robust dual topological character with spin-valley polarization in a monolayer of the Dirac semimetal Na_3Bi. *Phys. Rev. B* **95**, 075404 (2017).
3. Crisostomo, C. P. *et al.* Robust large gap two-dimensional topo- logical insulators in hydrogenated III-V buckled honeycombs. *Nano Lett.* **15**, 6568–6574 (2015).
4. See http://www.flapw.de.
5. Mostofi, A. A. *et al.* An updated version of wannier90: A tool for obtaining maximally-localised Wannier functions. *Comput. Phys. Commun.* **185**, 2309 – 2310 (2014).
6. Yang, Y. *et al.* Time-reversal-symmetry-broken quantum spin Hall effect. *Phys. Rev. Lett.* **107**, 066602 (2011).
